# Insulin-Like Growth Factor 1 Receptor as a Therapeutic Target in Ewing Sarcoma: Lack of Consistent Upregulation or Recurrent Mutation and a Review of the Clinical Trial Literature

**DOI:** 10.1155/2013/450478

**Published:** 2013-01-28

**Authors:** Alison O'Neill, Nilay Shah, Naamah Zitomersky, Marc Ladanyi, Neerav Shukla, Aykut Üren, David Loeb, Jeffrey Toretsky

**Affiliations:** ^1^Lombardi Comprehensive Cancer Center, Georgetown University Medical Center, Washington, DC 20057, USA; ^2^Department of Gastroenterology and Nutrition, Boston Children's Hospital, 300 Longwood Avenue Boston, MA 02115, USA; ^3^Department of Pathology and Human Oncology & Pathogenesis Program, Room S-801, Memorial Sloan-Kettering Cancer Center, 1275 York Avenue New York, NY 10065, USA; ^4^Department of Pediatrics, Memorial Sloan-Kettering Cancer Center, 1275 York Avenue, New York, NY 10065, USA; ^5^Division of Pediatric Oncology, Department of Oncology, The Sidney Kimmel Comprehensive Cancer Center, Johns Hopkins University, Baltimore, MD 21287, USA

## Abstract

The insulin-like growth factor 1 receptor (IGF-1R) has been considered an important therapeutic target in Ewing sarcoma (ES), generating a need to identify the subset of patients most likely to respond to IGF-1R inhibitors. We assessed IGF-1R expression in ES cell lines and patient tumors to understand the variable clinical responses to anti-IGF-1R therapy. Using ligand-binding displacement, we measured between 13,000 and 40,000 receptors per cell in ES cell lines. We used ELISA to quantify IGF-1R in patient tumors, which expressed 4.8%  ± 3.7 to 20.0%  ± 0.2 of the levels in a positive control cell line overexpressing IGF-1R. Flow cytometry showed markedly reduced IGF-1R expression in ES cell lines compared to a standard positive control cell line. The *IGF1R* gene was sequenced in 47 ES tumor samples and 8 ES cell lines; only one tumor sample showed a nonsynonymous mutation, R1353H, in a region with low functional impact. Finally, we assessed IGF-1R pathway activity in the ES stem cell (ESSC) population, to characterize its potential for resistance to anti-IGF-1R therapy, using Luminex technology. We found no significant differences in IGF-1R pathway activity between ESSCs and the total cell population. Overall, our findings suggest that IGF-1R as a therapeutic target in this sarcoma may require reevaluation.

## 1. Introduction

Ewing sarcoma (ES) is a malignancy of the bone and soft tissue that occurs predominantly between the ages of 3 and 40 and is characterized by a *t*(11; 22)(*q*24; *q*12) chromosomal translocation in 85% of cases, resulting in the oncogenic fusion protein EWS-FLI1. Despite aggressive multimodal treatment and advances in surgery, radiation, and chemotherapy, 30% of patients with localized disease at diagnosis and 75–80% of patients who present with metastases eventually die from their disease [1]. The poor prognosis for patients with ES indicates an urgent need for the development of targeted therapies.

The insulin-like growth factor 1 receptor (IGF-1R) has been the subject of more than 20 years of research as a potential therapeutic target in ES [2]. These investigations have included the role of IGF-IR in the initiation of ES [3], *in vitro *and *in vivo* effects of blocking IGF-IR [4–8], and the expression of signaling components in patients with ES [9–11]. As a result of these data, patients with ES were thought to be ideal candidates for therapy directed towards the IGF-1R axis. ES patients were thus enrolled in early clinical trials of humanized monoclonal antibodies against IGF-1R with the expectation of significant antitumor effects. The phase II studies showed objective response rates that ranged from 8 to 15%, with the vast majority being partial responses measured in weeks to months [12–14]. Anti-IGF-1R therapy clearly benefits a subset of patients, and it will be essential to find markers to identify the patients most likely to respond. 

The clinical interest in identifying those patients that might benefit from anti-IGF-1R therapy has prompted closer scrutiny of IGF-1R as a target. Other receptor tyrosine kinases (RTK) that have been successfully targeted contain either activating mutations or significant gene amplification [15, 16]. Previous studies have reported IGF-1R overexpression in ES, in support of pursuing IGF-1R targeted therapies for this disease [17]. However, comparison values are critical in reporting overexpression of a protein, and these values, as well as the thresholds that characterize clinically relevant overexpression, are often poorly defined. 

In order to understand the variable response to anti-IGF-1R therapy [10, 18], we sought to verify the expression levels of this receptor in ES. Additionally, we characterized the expression and activation of the IGF-1R signaling pathway in Ewing sarcoma stem-like cells (ESSCs), a population of tumor cells that are relatively resistant to chemotherapy [19, 20], in order to investigate a potential reservoir for resistance to anti-IGF-1R therapy. Finally, we critically review the literature on IGF-1R in ES, in an effort to shed light on the recently published outcomes of targeting the IGF-1 receptor in patients with Ewing sarcoma.

## 2. Materials and Methods

### 2.1. Cell Lines, Aldefluor Assay, and Fluorescence-Activated Cell Sorting

Established ES cell lines TC71, TC32, A4573, MHH-ES-1, RDES, 5838, and SK-N-MC were maintained in RPMI-1640 growth medium (Life Technologies, Carlsbad, CA) supplemented with 10% fetal bovine serum (Thermo Scientific, Logan, UT) and 1% HEPES (Life Technologies). Established Ewing sarcoma SKES cells were maintained in McCoy's 5A growth medium (Life Technologies) supplemented with 15% fetal bovine serum. NWTb3, an NIH-3T3 cell line overexpressing human wild-type IGF-1R, was maintained in Dulbecco's Modified Eagle Medium (Life Technologies) supplemented with 10% fetal bovine serum. All cell lines were maintained at 60–80% confluence in a humidified environment at 37°C containing 5% CO_2_. 

Ewing sarcoma stem-like cells (ESSCs) were selected for analysis from these cell lines using a previously validated cancer stem cell marker, aldehyde dehydrogenase (ALDH) [20]. Enrichment for this enzyme was assessed using the Aldefluor assay according to manufacturer's instructions (Stem Cell Technologies, Vancouver, BC). ALDH-high ES cells were selected by comparing Aldefluor-stained cells incubated with and without the ALDH inhibitor diethylaminobenzaldehyde (DEAB). Fluorescence-Activated Cell Sorting (FACS) was performed using the FACSAria cell sorter and FACSDiva software (BD Biosciences, Franklin Lakes, NJ). Nonviable and clumped cells were excluded based on light scatter properties and particle size. The brightest 2–5% of cells were collected as ESSCs/ALDH-high cells, and the dimmest 10–25% were collected as non-ESSCs/ALDH-low cells. Aldefluor-treated cells were passed through the FACS setup, but not sorted, to be used as the control condition referred to as “flow-through.”

### 2.2. IGF-1 Ligand-Binding Displacement Quantification of IGF-1R in ES Cell Lines

A4573, TC71, 5838, and SK-N-MC cells were incubated with 0, 1, 2, 5, 10, 20, 100, 200, or 2,000 ng/mL ^125^I-IGF-1 for 6 hours at 4°C, washed with PBS, and then incubated with 0.2N NaOH for 1 hour at 37°C. Bound cells were adsorbed onto filter paper and radioactivity was quantified using radioscintography.

### 2.3. Enzyme-Linked Immunosorbent Assay (ELISA) of IGF-1R

IGF-1R levels in ten patient tumor samples were measured by sandwich ELISA. Plates were coated with IGF-1, blocked with 5% milk, and then incubated with tumor sample lysates at 50 *μ*g protein per well, or with NWTb3 lysate as a positive control, in 1% BSA in PBS. IGF-1R binding was detected using *α*IR3 antibody at 1 *μ*g/mL in 1% BSA in PBS and anti-mouse secondary antibody. Standard curves were created using 0.1 *μ*g to 30 *μ*g NWTb3 lysate.

### 2.4. Flow Cytometric Analysis

 ES cells were stained with Aldefluor and/or IGF-1R*α* (1H7): sc-461 antibody (Santa Cruz Biotechnology, Santa Cruz, CA) according to the manufacturer's instructions. The dual-stained cells were then analyzed by flow cytometry using the FACSAria cell sorter and FACSDiva software to assess levels of membrane-associated IGF-1R relative to ALDH enrichment.

In bicolor analyses of IGF-1R and ALDH expression, ALDH-high cells were defined as the brightest 5% of Aldefluor-stained cells, and ALDH-low cells were the remaining 95% of cells, except where less than 5% of cells were stained brighter than the Aldefluor-blocked DEAB control, in which case the gate was set at 0.1% or less of the DEAB control. The gate for IGF-1R expression was set at 0.1% or less of unstained control cells. Antibody-labeled cells brighter than this threshold were defined as IGF-1R positive, and cells below this threshold were defined as IGF-1R negative.

### 2.5. Luminex xMAP Analysis

ALDH-high, ALDH-low, and unsorted SKES and TC71 cells were plated at 500,000 cells per well in collagen-coated 24-well plates in their appropriate growth medium with 10% FBS plus 1% penicillin/streptomycin. Plates were incubated for 6 hours at 37°C and 5% CO_2_ to allow cells to adhere, and then growth medium was replaced with serum-free DMEM to allow cell signaling to become quiescent. After incubating cells for additional 6 hours at 37°C and 5% CO_2_, cell lysates were collected and stored at −80°C. 

The Luminex 100 system (Luminex Corporation, Austin, TX) was used to quantify IGF-1R, Akt, GSK-3*β*, IR, IRS-1, p70S6K, and PRAS40 from cell lysates of two separate experiments per cell line in duplicate, according to manufacturer's instructions. Total and phosphorylated proteins were assessed using separate multiplex assays for the IGF-1R signaling pathway according to the manufacturer's instructions: Akt Phospho 7-Plex Panel (LHO0001) and Akt Total 7-Plex Panel (LHO0002, Life Technologies, Carlsbad, CA). Phosphorylation sites detected for each protein were the following: IGF-1R [pYpY1135/1136], Akt [pS473], GSK-3*β* [pS9], IR [pYpY1162/1163], IRS-1 [pS312], p70S6K [pTpS421/424], and PRAS40 [pT246].

### 2.6. Quantitative Reverse Transcription Polymerase Chain Reaction

RNA was extracted from ALDH-sorted and flow-through TC71 cells using the PicoPure RNA Isolation Kit (Life Technologies, Carlsbad, CA) according to manufacturer's instructions. Isolated RNA was reverse transcribed using SuperScript VILO (Life Technologies) according to manufacturer's instructions. For quantitative PCR, 5 *μ*L cDNA was combined with 12.5 *μ*L SYBR Green SuperMix and appropriate primers, and qPCR was performed using a standard two-step amplification/melt protocol on a Mastercycler Realplex qPCR system (Eppendorf, Hauppauge, NY), and results were analyzed using the 2^−ΔΔCt^ method.

The following primer sequences were designed across exon-exon junctions using Primer 3 Plus in order to detect the respective mRNA transcripts: IGF-1 forward: 5′-GGAGCTGTGATCTAAGGAGGC-3′; IGF-1 reverse: 5′-GGACAGAGCGAGCTGACTT-3′; ALDH1A1 forward: 5′-TGGCTTATCAGCAGGAGTGTT-3′; ALDH1A1 reverse: 5′-CACTTACCACGCCATAGCAA-3′. IGF-1R and IR primer sequences have been described [21]. Levels of mRNA were normalized to the 18S transcript, using previously described primer sequences [22].

### 2.7. PCR Amplification and DNA Sequencing of IGF1R

All coding exons of *IGF1R* were amplified as amplicons of 500 bp or less, covering the exonic regions plus at least 50 bp of intronic sequences. M13 tails were added to the primers to facilitate Sanger sequencing. PCR reactions were carried out in 384-well plates. Templates were purified using AMPure (Agencourt Biosciences, Beverly, MA). The purified PCR reactions were split into two and sequenced bidirectionally with M13 forward and reverse primer and Big Dye Terminator Kit v.3.1 (Applied Biosystems, Foster City, CA), at Agencourt Biosciences. Dye terminators were removed using the CleanSEQ kit (Agencourt Biosciences), and sequence reactions were run on ABI PRISM 3730xl sequencing apparatus (Applied Biosystems, Foster City, CA). Mutations were detected using an automated detection pipeline by the MSKCC Bioinformatics Core. 

### 2.8. Statistical Analyses

For all studies showing statistical analyses, 2-tailed Student's *t*-tests for independent samples were performed using Prism 4 software (GraphPad, Inc.). *P* < 0.05 was considered statistically significant.

## 3. Results

### 3.1. Quantification of IGF-1R in ES Cell Lines and Patient Tumors

Our experiments evaluated four different techniques to measure IGF-1R expression levels in ES cell lines and patient tumors including ligand-binding displacement, sandwich ELISA, immunohistochemistry, and flow cytometry. In order to define IGF-1R as a therapeutic target in ES, we first measured absolute expression of the IGF-1 receptor in ES cell lines using radiolabeled IGF-1 ligand-binding displacement. This analysis demonstrated a range of ~13,000 to ~40,000 receptors per cell in ES, with minimum expression in A4573 and maximum expression in SK-N-MC (Table 1).

**Figure 1:**
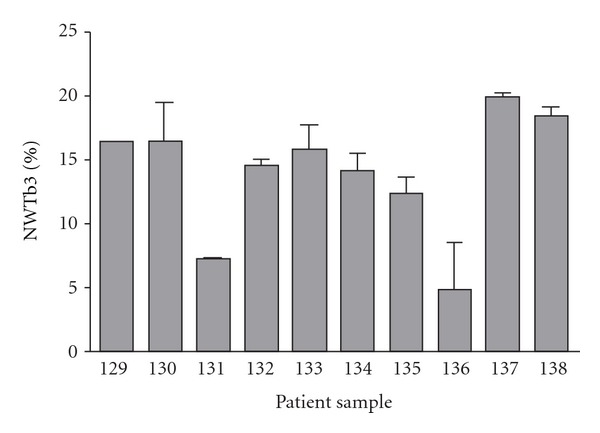
Quantification of IGF-1R in patient tumor samples by sandwich ELISA, detected using *α*IR3 antibody and compared to NWTb3 positive control.

We then sought to quantify IGF-1R in ES patient tumors from a previous study in which IGF-1R had been evaluated by immunohistochemistry [23]. We assessed IGF-1R in ten tumor samples by ELISA, using NWTb3 cell lysate as a standard. The IGF-1R content of these patient samples ranged from 4.8% ± 3.7 to 20.0% ± 0.2 of levels present in NWTb3 lysates (Table 2, Figure 1). Between 5% and 100% of cells in each tumor sample were positive for IGF-1R when previously assessed by immunohistochemistry (Table 2) [23].

**Figure 2:**
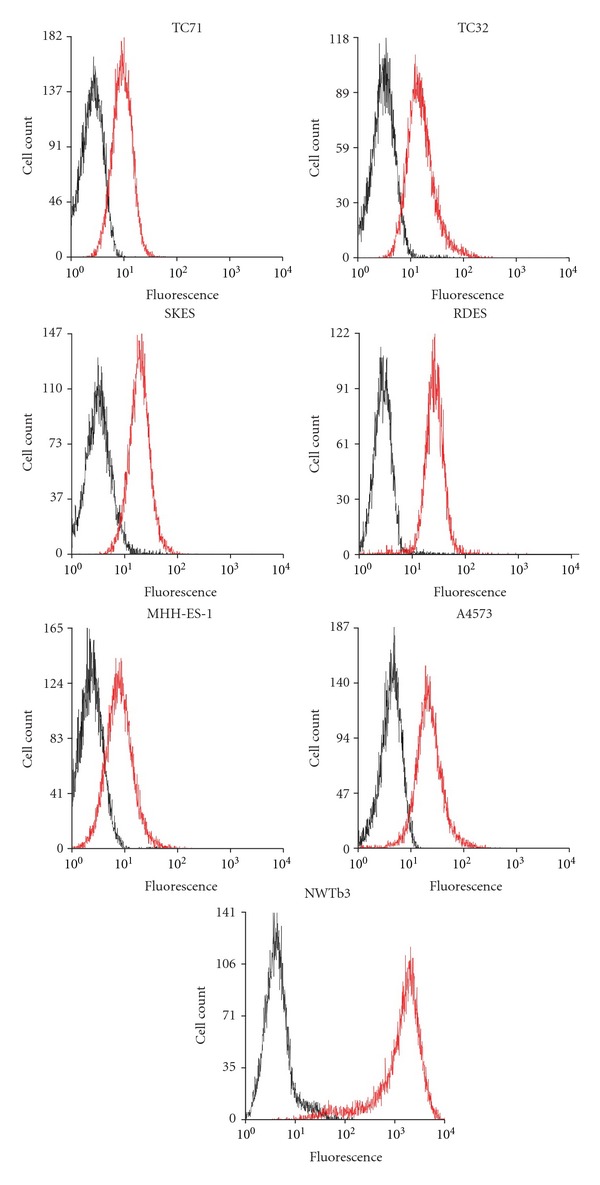
Flow cytometric analysis of IGF-1R-PE-labeled ES and NWTb3 cells. Black: unlabeled cells; Red: IGF-1R-PE-labeled cells.

In order to evaluate the degree of IGF-1R overexpression in Ewing sarcoma cells, we assessed relative IGF-1R surface expression in ES cell lines and NWTb3 cells, the NIH-3T3 cell line transfected to overexpress human IGF-1R, using flow cytometry. NWTb3 cells showed significantly greater brightness than ES cells after incubation with a phycoerythrin-conjugated IGF-1R antibody (IGF-1R-PE). NWTb3 cells showed a mean fluorescence of 1127.32, comparatively greater than the mean fluorescence of TC71 ($\overset{-}{x}=14.59$), RDES ($\overset{-}{x}=22.57$), SKES ($\overset{-}{x}=20.57$), MHH-ES-1 ($\overset{-}{x}=8.45$), A4573 ($\overset{-}{x}=21.60$), and TC32 ($\overset{-}{x}=13.53$) cells (Figure 2).

### 3.2. Assessment of IGF-1R Pathway Levels in ALDH-Sorted ES Populations

**Figure 3:**
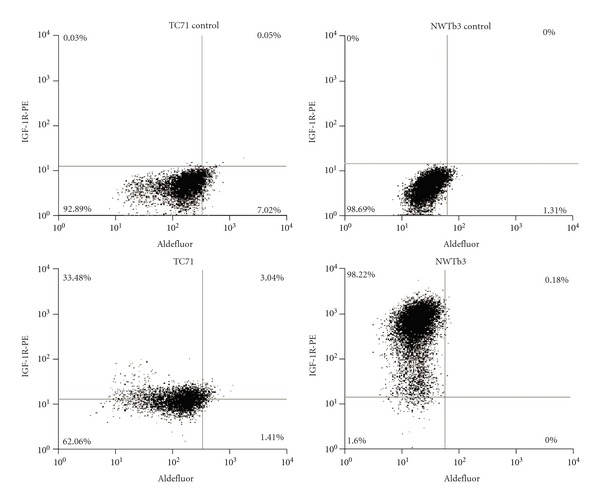
Gating of cells for flow cytometric analysis of IGF-1R surface expression in TC71 and NWTb3 cells stained with Aldefluor. Top: Aldefluor alone without IGF-IR-PE antibody. Bottom: Aldefluor plus IGF-1R-PE antibody.

**Figure 4:**
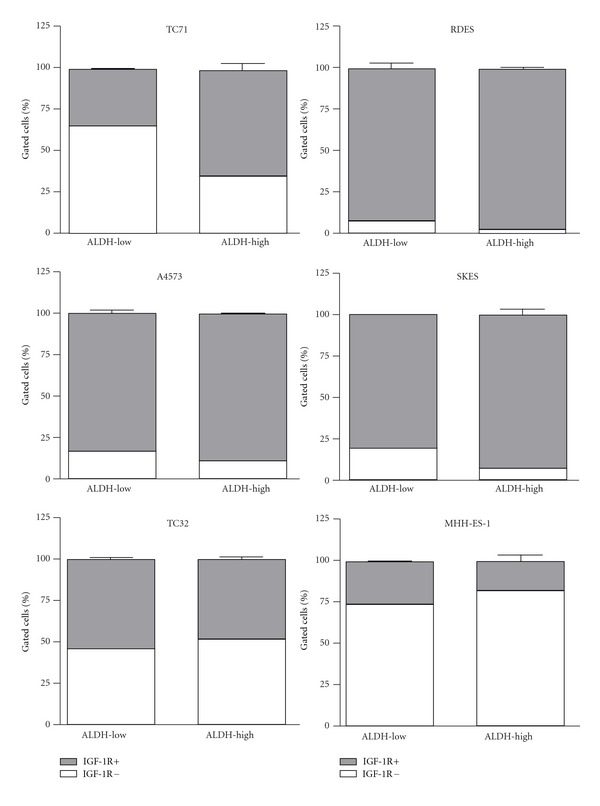
IGF-1R surface expression in ALDH-gated ESFT populations by cell line. Error bars represent SEM of duplicate measurements.

Since absolute IGF-1R levels are relatively low in ES cell lines, we sought to determine whether Ewing sarcoma stem-like cells constituted a population of cells resistant to anti-IGF-1R therapy based upon lower expression of IGF-1R than the total population. We stained cells for ALDH expression using the Aldefluor assay and labeled them with IGF-1R-PE antibody in order to assess the amount of surface IGF-1R in relation to ESSC identity in each individual cell. The 5% of cells demonstrating brightest Aldefluor staining were gated as ALDH-high, and the remaining 95% of cells were considered ALDH-low in order to compare ESSCs with the total population of ES cells. Gating of IGF-1R expression was set at 0.1% or less of the brightness of unlabeled cells, and all cells above that threshold were considered IGF-1R-positive (Figure 3). Three of six ES cell lines showed significantly different levels of IGF-1R in ALDH-high and ALDH-low cells, with TC71 and A4573 demonstrating higher IGF-1R expression in ESSCs and TC32 showing lower IGF-1R expression in this population compared to unsorted cells (Figure 4).

**Figure 5:**
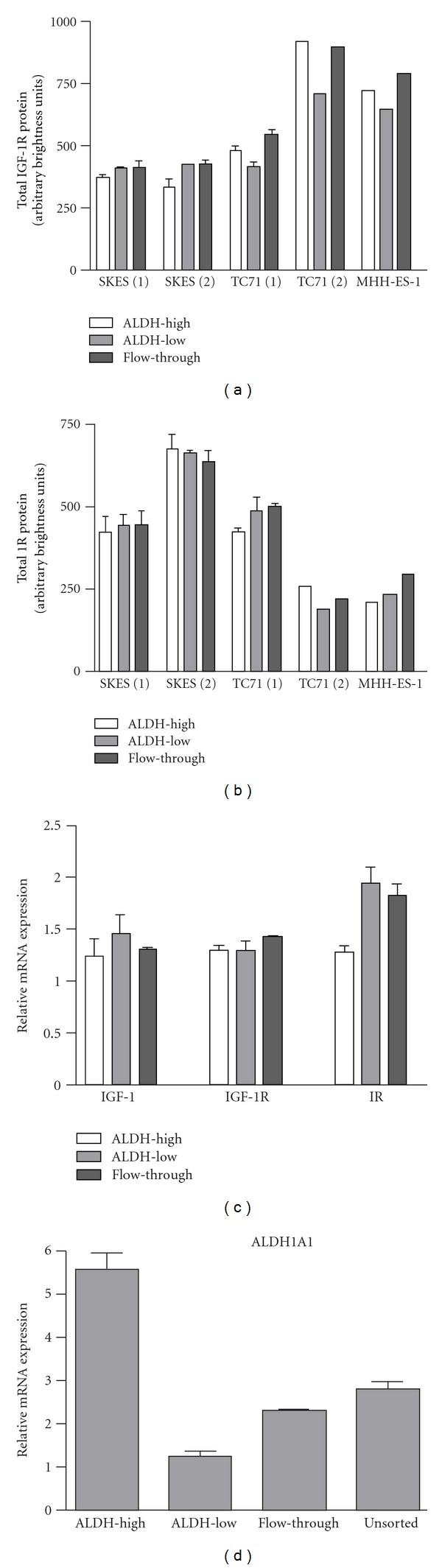
Total expression of IGF-1R pathway members in ES cell lines. (a) Luminex xMAP quantification of total IGF-1R protein in ALDH-sorted ES cells. (b) Luminex xMAP quantification of total IR protein in ALDH-sorted ES cells. (c) qRT-PCR quantification of IGF-1, IGF-1R, and IR mRNA transcripts in ALDH-sorted TC71 cells. (d) Quantification of ALDH1A1 mRNA in Aldefluor-sorted TC71 cells. Numbers in parentheses refer to replicate experiments.

A second method measured IGF-1 receptor levels in ALDH-sorted ES cell lysates using the Luminex xMAP antibody-based protein quantification assay. Differences in IGF-1R total protein levels among ALDH-sorted ES cell populations were not statistically significant, though ALDH-high SKES cells tended to express slightly less IGF-1R than ALDH-low or unsorted cells, while ALDH-high TC71 cells expressed slightly more IGF-1R than respective ALDH-low or unsorted populations (Figure 5(a)). Total amounts of Insulin Receptor (IR) did not vary significantly or consistently among ALDH-sorted populations but did demonstrate variability among cell lines and repeated experiments (Figure 5(b)). Total levels of Akt, GSK-3b, IRS-1, p70S6K, and PRAS40 similarly showed no significant differences in expression among ALDH-sorted cells (data not shown). Quantification of phosphorylated proteins revealed no significant differences in baseline phosphorylation of Akt pathway members in ESSCs (data not shown). 

Using a third method, we quantified mRNA transcripts of IGF-1, IGF-1R, and IR using qRT-PCR. Again, no significant differences in IGF-1 or IGF-1R expression were identified among ALDH-sorted TC71 cells (Figure 5(c)). Elevated ALDH1A1 expression in Aldefluor-sorted TC71 cells was confirmed by qRT-PCR (Figure 5(d)). 

### 3.3. Screening for IGF1R Mutations in ES Tumors and Cell Lines

All coding exons of *IGF1R* were subjected to Sanger sequencing in DNAs from 47 tumor samples of Ewing sarcoma (all fusion verified) and 8 Ewing sarcoma cell lines (TC71, A4573, TC32, SK-PN-DW, SK-ES-1, RDES, A673, and CHP100). Of these 55 total DNAs, only one sample, an ES tumor sample, showed a nonsynonymous mutation, R1353H, encoded in exon 21, the last exon. This poorly conserved residue is 3′ to the kinase domain and the mutation is predicted to have a low functional impact (http://mutationassessor.org/). 

## 4. Discussion of Current Results

ES is an aggressive cancer and prognosis remains particularly poor for patients with metastatic or recurrent disease, hence the need for new specific and targeted therapies for ES. Extensive preclinical studies reporting the efficacy of IGF-1R inhibitors in ES led to the development of several humanized monoclonal antibodies against IGF-1R, most of which have completed phase I and phase II clinical trials in patients with ES. Though these treatments are generally well tolerated, the results of the phase II trials that have been published to date have fallen short of expectations, with 8–15% of patients demonstrating objective response to therapy [12–14]. We assessed the expression of IGF-1R in the total population of several ES cell lines and patient samples as well as in Ewing sarcoma stem-like cells (ESSCs), with the intention of investigating this population as a possible source of resistance to anti-IGF-1R therapy.

While our results support previous data that ES cell lines express the IGF-1 receptor, our findings suggest that IGF-1R levels are low compared to cell lines transformed by IGF-1R [17]. Ligand-binding displacement analysis showed that ES cell lines express between 13,000 and 40,500 IGF-1 receptors per cell; in an early report investigating the role of IGF-1R in transformation of mouse fibroblasts, wild type NIH-3T3 cells expressed 65,000 receptors per cell yet did not demonstrate anchorage independent growth. NWTb3 cells expressing 972,000 receptors per cell, however, reliably demonstrated colony formation in soft agar [24]. Another recent report has demonstrated low IGF-1R levels in clinical samples, citing expression in only 20% of cases [18]. Our analysis of ES cell lines by flow cytometry demonstrated that IGF-1R is expressed at relatively low levels, around two-log-fold lower than the levels detected in NWTb3 cells. Furthermore, our assessment of IGF-1R expression in patient tumors by ELISA revealed low levels of the receptor in comparison with NWTb3 cells, showing *α*IR3 binding between 4% and 20% of the positive control. Analysis of these tumors by immunohistochemistry showed the presence of IGF-1R on 5% to 100% of cells in each sample. However, IGF-1R levels detected by ELISA did not reliably correlate with receptor levels measured by IHC. While both methods demonstrate the variability of IGF-1R expression in clinical specimens of ES, the lack of correlation in the results underscores the important difficulties of quantifying biological markers in patient tumors. In light of these observations, further efforts are warranted to clarify the claims that IGF-1R is overexpressed in Ewing sarcoma, and that ES cells are truly addicted to IGF-1R signaling.

Cancer stem cells are thought to play a role in the relapse of disease after an initial response to therapy. In order to understand the number of ES patients on phase II trials of IGF-1R antibodies whose initial response to therapy was followed by progressive disease [12–14], we sought to evaluate IGF-1R expression in Ewing sarcoma stem-like cells to assess this population's potential for resistance to IGF-1R-targeted therapy. Since the lower proliferative rate of ESSCs is implicated in their resistance to chemotherapy, we hypothesized that this characteristic might also be associated with a lower level of IGF-1R expression, and therefore, a lower level of dependence on the activity of this pathway. Although we observed significant differences in IGF-1R expression among these populations in three of six ES cell lines using flow cytometry, the absolute differences in expression are most likely not great enough to indicate a difference in the function of the IGF-1R pathway in ESSCs. Furthermore, we did not identify consistent differences in expression of IGF-1, IGF-1R, or IR in ESSCs using other protein quantification methods, and thus it appears unlikely that this population is involved in mechanisms of resistance to IGF-1R therapy. Although ESSCs tend to express slightly higher levels of these members of the IGF-1 system, the differences are not large enough to indicate that they would have increased susceptibility to IGF-1R inhibition.

Our findings contribute to a growing body of work that calls into question the longstanding high hopes for anti-IGF-1R therapy in light of the actual nature of the IGF-1 receptor's role in Ewing sarcoma biology. Although a substantial number of preclinical studies support an important role of the IGF-1 receptor in the tumorigenesis and continued survival of ES, IGF-1R lacks the strong characteristics of other tyrosine kinases successfully targeted in cancer, such as HER2, which is often expressed at levels reaching 2 million receptors per cell as a result of gene amplification in HER2-positive breast cancer [15], and BCR-ABL, a fusion protein resulting in a constitutively active tyrosine kinase in chronic myelogenous leukemia [16]. 

Mutations in IGF-1R have not yet been identified in sarcoma in the published literature. A limited set of tumors analyzed for IGF-1R mutations are described in the Sanger COSMIC database, and mutations were identified in 13 of 1486 tumor samples (0.9%) within 7 of 18 tumor types. Of these mutations, about half are distal to the kinase domain and half are proximal, and no mutations within the kinase domain were identified. It is unclear whether these mutations would have a functional effect based on their location within the gene [25]. These prior sequencing data are consistent with our findings reported here, with only 1 of 55 Ewing sarcoma tumors or cell lines bearing a nonsynonymous mutation of low predicted functional impact. Importantly, a recent study of the IGF-1R system as a prognostic indicator in Ewing sarcoma reported that, contrary to prior assumptions, high expression of IGF-1 and IGF-1R predicted higher rates of event-free and overall survival, suggesting that the patients most in need of targeted therapies may not be in the subset of patients who will respond to IGF-1R treatment [11].

## 5. Early Development of IGF-1R as a Target for Therapy in ES

The promise of IGF-1R inhibition as a treatment for Ewing sarcoma garnered remarkable enthusiasm over the course of the past two decades. The first study to identify IGF-1R in ES reported the expression of IGF-1 and IGF-1R in ES tumors and demonstrated *in vitro* growth inhibition using the anti-IGF-1R antibody *α*IR3 [2]. This report led to a number of other studies over the following decade demonstrating the importance of the IGF-1 system, and the potency of its inhibition, in ES [3, 17, 26–29]. However, in these early *in vitro* studies, growth inhibition occurred due to cytostatic, rather than cytotoxic, effects as demonstrated by a moderate inhibition of proliferative rate and a modest induction of apoptosis [17]. This distinction, among others, is critical in assessing the results of recently published and ongoing clinical trials in patients with advanced and refractory Ewing sarcoma.

The first study investigating the efficacy of the *α*IR3 antibody *in vivo* reported prevention of tumor formation in 56% of athymic mice injected with ES cells [4]. Successful inhibition of tumor growth was subsequently demonstrated in ES xenograft models with dominant negative receptors, antisense knockdown, and small molecules targeting IGF-1R [6, 7, 30]. It is important to note that in the antisense and dominant negative IGF-1R experiments, tumor cell expression of IGF-1R was impaired at the time of injection, thus preventing robust establishment of tumors, and in the *α*IR3 and small molecule studies, treatment also began before tumors were well established. Although the tumor inhibition reported in these studies warranted further investigation of targeted therapies, these experiments modeled treatment at the very onset of tumor formation and therefore do not speak directly to the potential activity of anti-IGF-1R treatments in established or advanced human disease.

The Pediatric Preclinical Testing Program (PPTP) reported mixed results of anti-IGF-1R treatments developed for clinical use in Ewing sarcoma xenograft models; these were evaluated with larger starting tumor volumes, between 200 and 500 mm^3^ [31]. Monoclonal antibody SCH 717454 (Schering-Plough) showed significant improvement in event-free survival for 2 of 5 ES xenografts, including one complete response [32]. The monoclonal antibody cixutumumab (IMC-A12; ImClone Systems) induced an intermediate response in only 1 of 5 ES xenografts, where the activity was attributed primarily to growth inhibition rather than tumor regression [33]. At the time these preclinical studies were published, early clinical trials of anti-IGF-1R therapy were already under way in patients with Ewing sarcoma.

## 6. Clinical Trials Evaluated Both IGF Components and Clinical Response to IGF-1R Antibodies

**Figure 6:**
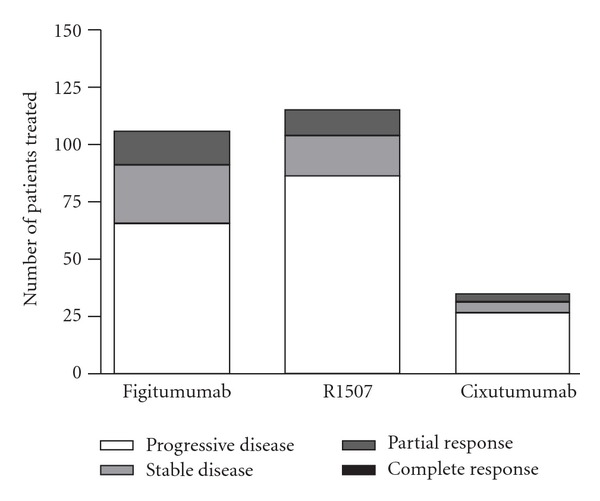
Clinical results of anti-IGF-1R therapy: response to therapy in phase II trials for Figitumumab, R1507, and Cixutumumab.

Currently, phase I/II or phase II trials of three humanized monoclonal antibodies have been conducted in Ewing sarcoma: R1507 (Roche, Basel, Switzerland), figitumumab (CP-751871; Pfizer, New London, CT), and cixutumumab (IMC-A12; ImClone systems, Branchburg, NJ). Among the phase II cohorts, figitumumab showed the highest objective response rate of 14.2%, where R1507 showed a response rate of 9.6% and cixutumumab demonstrated the lowest rate of 8.6% (Figure 6) [12–14]. The need for the identification of markers to predict at diagnosis who will belong to the small subset of patients that respond to these therapies has already been stated by many, including the authors of these studies.

Several members of the IGF-1 system have been identified as potential prognostic indicators in ES, most notably IGF-1 and IGFBP-3 [34]. An important factor in support of targeting IGF-1R in ES is that EWS-FLI1 acts to suppress transcription of IGFBP3 in tumor cells, thereby resulting in higher levels of circulating IGF-1 and thus increasing activation of the IGF-1R pathway [35]. Indeed, high IGFBP3, low IGF-1, and high IGFBP-3/IGF-1 ratios were associated with better overall survival in a retrospective study of patients with ES [9]. However, a more recent prospective study failed to confirm the correlation between the IGFBP-3/IGF-1 ratio and overall survival [10]. Furthermore, another recent publication counters the widespread assumption that higher expression of the IGF-1 ligand and its receptor correlate with more aggressive tumors and instead reports that higher levels of these proteins indicate better event-free and overall survival [11]. Together, these analyses again suggest that although the IGF-1 system clearly plays a role in ES tumor biology, neither high ligand availability nor high receptor number is consistently defined as a predominant driving factor for poor patient outcomes.

Anti-IGF-1R therapy clearly provides therapeutic benefit for a select group of patients, and identifying predictive markers of this effect would be an extraordinary advance toward individualizing treatment. There have been several efforts thus far to characterize the IGF-1R pathway in clinical trial patients in order to identify such potential markers. In the cixutumumab trial, patients were evaluated for tumor levels of IGF-1, IGF-2, and IGF-1R by immunohistochemistry, and the study reported no apparent correlation between expression of these three proteins and response to cixutumumab treatment [14]. In the figitumumab trial, intermediate pretreatment IGF-1 levels were associated with higher overall survival, with patients in the second and third quartiles of baseline IGF-1 levels showing longer median survival probabilities by several months when compared to those in the highest and lowest quartiles [12]. Lastly, the results from the R1507 trial report that high baseline IGF-1 levels correlate with better overall survival but not with rate of response to treatment [13]. As has been recently observed, these trends suggest that high IGF-1 may be an overall indicator for better prognosis rather than a marker for response to anti-IGF-1R therapy [36].

## 7. Combination Therapy with IGF-1R Inhibitors

There is evidence to suggest that IGF-1R pathway inhibition significantly increases or synergizes with the toxic effects of chemotherapy *in vitro*, with combination of *α*IR3 treatment and either doxorubicin or vincristine demonstrating particularly dramatic results [5, 29, 37, 38]. Furthermore, simultaneous inhibition of IGF-1R and various members of the IGF-1R pathway, or other surface receptors such as IR, c-KIT, or EGFR, presents another enticing prospect for combination therapy. Studies have shown potential positive interactions of IGF-1R inhibitors with the c-Kit inhibitor imatinib [38] and the Her2 inhibitor trastuzumab [39]. In addition, PDGFR and FGFR have been identified as potential targets in ES [40, 41]. Inhibiting these along with IGF-1R may provide additional benefit.

Rapamycin is an inhibitor of mammalian target of rapamycin (mTOR) complex 1, a downstream signal transducer of IGF-1R, and has been extensively investigated in combination with IGF-1R inhibition in ES. The combination of rapamycin and cixutumumab demonstrated synergistic antitumor effects *in vitro *and* in vivo* in a Stage 2 study by the Pediatric Preclinical Testing Program (PPTP) [42, 43]. However, results of these combination studies have not been entirely consistent. Six Ewing sarcoma xenograft models treated with figitumumab and rapamycin showed highly variable responses [44]. In another study, rapamycin enhanced the activity of the IGF-1R inhibitor ganitumab (AMG-479; Amgen) *in vitro* and *in vivo*, though this combination also resulted in increased IR signaling [45].

As a result of these findings, inhibitors of mTOR have begun to be included in clinical trials of anti-IGF-1R antibodies. In the phase I/II study of figitumumab, rapamycin was added to figitumumab as a salvage therapy, but no objective responses were reported [12]. In the phase I study of cixutumumab, the mTOR inhibitor temsirolimus was added to the regimen in a subset of patients. Five (29%) of ES patients had tumor regressions of over 20%, including one complete response [46]. Thus, emerging evidence suggests that combination therapy may benefit a subset of patients compared to IGF-1R inhibition alone, and the magnitude of this effect may depend on the specific IGF-1R inhibitor.

However, despite the numerous potential candidates for combination therapy with anti-IGF-1R antibodies, it is necessary to take into account the mixed results of preclinical and clinical studies as well as the modest biological evidence underlying IGF-1R as a target for Ewing sarcoma therapy. Before committing further resources to combination clinical trials, predictable and robust *in vivo *responses should be sought. There is no mouse model of ES in which a response to a given treatment provides a confident correlation with efficacy in humans. However, no agent without a robust response in an ES xenograft has yet provided a clinical breakthrough, an observation that underlies the PPTP testing guidelines [31]. As the relatively low response rates in the anti-IGF-1R clinical trials suggest, more rigorous standards for evaluation of preclinical data may be warranted before further strategies targeting the IGF-1 system should be moved into the clinic.

## 8. Conclusions

Valuable insights have been made into the role of the IGF-1R pathway in ES biology throughout the past two decades. However, in reexamining the body of work on IGF-1R and ES in light of the clinical trials published to date, as well as new data assessing IGF-1R levels in ES cell lines and patient tumors, patterns are emerging that help us to understand the limited activity of this strategy in patients with this disease. Although further development of approaches to target this complex system may be warranted, it is evident that targeting the IGF1R is not, as was initially hoped, a straightforward solution to effectively treat this aggressive malignancy. 

## Figures and Tables

**Table 1:** Ligand-binding displacement quantification of IGF-1R surface expression in ES cell lines. The column adjacent to cell line names refers to replicate experiments.

Cell Line		*K*_*d*_ (M)	Receptor number per cell
A4573	1	2.80 × 10^−10^	16895
	2	1.81 × 10^−10^	13163
ES-5838	1	2.69 × 10^−10^	27274
	2	2.23 × 10^−10^	14095
SK-N-MC	1	3.24 × 10^−10^	27519
	2	4.58 × 10^−10^	40424
TC71		2.37 × 10^−10^	25156

**Table 2:** ELISA and IHC quantification of IGF-1R in patient tumor samples.

Sample	*α*IR3 binding (% of NWTb3 ± SD)	IGF-1R IHC (% positive cells)
129	16.5 ± 0	100
130	16.5 ± 3.0	99
131	7.3 ± 0.2	100
132	14.6 ± 0.4	95
133	15.8 ± 1.9	100
134	14.2 ± 1.3	20
135	12.3 ± 1.3	10
136	4.8 ± 3.7	5
137	20.0 ± 0.2	40
138	18.6 ± 0.6	40
